# Standard Operating Procedure for Specimen Collection, Packaging and Transport for Diagnosis of SARS-COV-2

**DOI:** 10.31729/jnma.5260

**Published:** 2020-08-31

**Authors:** Lok Bahadur Shrestha, Khilasa Pokharel

**Affiliations:** 1 Department of Microbiology and Infectious Diseases, B.P. Koirala Institute of Health Sciences, Dharan, Nepal; 2 Department of Microbiology, Kathmandu Medical College, Sinamangal, Kathmandu, Nepal

**Keywords:** *nasopharyngeal swab*, *SARS-CoV-2*, *sop*

## Abstract

Coronavirus disease-19 (COVID-19) is caused by severe acute respiratory syndrome coronavirus-2 (SARSCoV-2). Specimen quality, and proper transportation is vital for accurate diagnosis. This standard operating procedure is designed to educate the clinicians, nurses, paramedics, and laboratory personnel regarding proper methods of sample collection, packaging, and transportation. Nasopharyngeal swabs and/or oropharyngeal swabs should be collected for real-time quantitative polymerase chain reaction to detect SARS-CoV-2. The sample should be collected wearing proper personal protective equipment, packed in a triple packaging system, and transported maintaining cold chain.

## INTRODUCTION

Coronavirus disease-19 (COVID-19) is caused by severe acute respiratory syndrome coronavirus-2 (SARS-CoV-2). Upon sequencing, the genomics of this novel coronavirus was found to share 79.5% homology to the genetic sequence of the SARS-CoV and therefore, the International Committee of Taxonomy of Viruses, renamed it as SARS-CoV-2. Meanwhile, the disease caused by the virus was named COVID-19 (Coronavirus disease 2019) by World Health Organization (WHO). WHO declared that COVID-19 should be characterized as pandemic on March 11, 2020. The clinical manifestation of COVID-19 includes fever, dyspnea, fatigue, dry cough, myalgia, headache, and loss of taste or smell. For severe and critical cases, patients suffer from acute respiratory distress syndrome, respiratory failure, and other serious complications including death. Within 5-7 days of infection, patients with COVID-19 demonstrate high viral load in upper respiratory specimens.^1^ Real-time reverse transcriptase-polymerase chain reaction (rRT- PCR) of nasopharyngeal and/or oropharyngeal swab has been considered the gold standard for diagnosis.^2–4^ Coronaviruses have a number of molecular targets withing their positive-sense, single-stranded RNA genome that can be used for PCR assays. These include structural proteins, including envelope glycoprotein spikes (S), envelope (E), transmembrane (M), helicase (Hel) and nucleocapsid (N). In addition to these genes that encode structural proteins, there are species-specific accessory genes that are required for viral replication. These include RNA-dependent RNA polymerase (RdRp), hemagglutinin-esterase (HE) and open reading frames ORF1a and ORD1b. Centre for disease control and prevention (CDC) recommends two nucleocapid protein targets (N1 and N2) while WHO recommends first line screening with E gene assay followed by a confirmatory assay using RdRp gene. Specimen quality, and maintenance of cold chain during transportation is vital for accurate result. False negative results occur due to improper timing of specimen collection and shortcoming in sampling proficiency, especially of nasopharyngeal swabs.^4–6^ Collecting the proper respiratory tract specimen at the right time from the right anatomic site is essential for a prompt and accurate molecular diagnosis of COVID-19. Appropriate measures are required to keep laboratory staff safe while producing reliable test results. Hence, this standard operating procedure (SOP) is designed to educate the clinicians, nurses, paramedics, and laboratory personnel regarding proper method for sample collection, packaging, and transportation.

## SPECIMENS TO BE COLLECTED

The performance of PCR depends upon several factors such as sample type, different stage of infection in patient, the skill of the collection, and the quality and consistency of the PCR assays being used. A nasopharyngeal specimen is the preferred choice for swab-based SARS-CoV-2 testing, but oropharyngeal sample should also be collected whenever feasible.^7,8^ Collecting both NP and OP swab has shown to improve sensitivity by upto two-fold. Sputum, bronchoalveolar lavage, and endotracheal aspirate specimens have higher sensitivity, but not collected routinely due to invasiveness of procedure and inconvenience.^6,8^ In case of patients presenting with acute respiratory distress, and requiring intubation, lower respiratory sample should be collected during intubation.^1,9^ If personal protective equipment can't be utilized due to scarcity of such PPE, other means of collecting upper respiratory tract specimens will be needed. One alternative option for collecting an upper respiratory tract specimen to evaluate patients with suspected COVID-19 is a self-collected saliva specimen. Repeated testing may be particularly be important if a patient has a clinical picture of viral pneumonia, a potential exposure history, and/or radiographic findings consistent with COVID-19 pneumonia.

## EQUIPMENT AND PREPARATION

Viral transport medium (VTM), swab sticks, ice-box, ice-pads, tongue depressor, marker, requisition form, and personal protective equipment (PPE) are needed beforehand. VTM contains 3 ml fluid composed of gelatin and antimicrobial agents in a buffered salt solution. It helps to prevent the specimen from drying, maintains the viability of the virus, andavoids the growth of contaminants. Swabs should be made up of rayon or dacron with plastic shaft. Cotton or calcium alginate swabs should not be used since it may inhibit PCR reaction. Stick with wooden shaft may cause trauma during sample collection. Ice-box and ice-pads are required for maintaining cold-chain during sample transportation. The ice-pads should be filled with water and stored in the freezer (-20°C) after before and after use.

Before beginning the procedure, the requisition form should be filled properly with patient details, travel history, contact history, and contact number of patient and the doctor. A proper PPE comprising of N95 mask, face shield, gown, goggles, shoe cover, and cap should be worn since sample collection is an aerosol-generating procedure. It is essential that we follow the pertinent respiratory and contact precautions certified by the Centres for Disease Control and Prevention and by our own institution and that we put on the PPE correctly. If possible, we should put on and take off the PPE in the presence of an observer to make sure there are no breaks in technique that may pose a risk of contamination.^7,8,10,11^

## PROCEDURE TO COLLECT NASOPHRYNGEAL SWAB

Firstly, we need to ask the patient to take off their mask and blow the nose into a tissue to clear the nasal passage. Then, slowly tilt the patient's head back slightly to access the nasal passage. We need to inform the patient to close their eyes to mitigate the displeasure of the procedure. After that, gently insert the swab along the nasal septum, parallel to the floor of the nasal passage, until resistance is felt. The swab should be left in place for few seconds to absorb secretions and then slowly remove while rotating it. In order to properly obtain an NP swab specimen, the swab must be inserted deeply into the nasal cavity and should elicit “tears”. Patients will likely flinch, but that means the swab has hit the target. Finally, the swab should be placed in the VTM and shaft should be broken.^12^

## PROCEDURE TO COLLECT OROPHRYNGEAL SWAB

For collecting oropharyngeal swabs, we need to ask the subject to open his or her mouth wide open. The tongue should be depressed using a tongue depressor and the posterior pharynx behind the tonsils should be swabbed, avoiding the tonsils. The OP swab should elicit a gag reflex, but there is much person to person variability in the gag response. Swabs should be kept in place for 10 seconds while twirling the swab three times. The collected swab should be placed into the same VTM and shaft needs to be broken. Swabs should have flocked non-toxic synthetic fibers such as polyester as well as synthetic nylon handles. Collecting a NP/OP swab specimen may carry a theoretical risk of transmitting theSARS-CoV-2 particularly if airborne transmission should be demonstrated as the investigation of the COVID-19 outbreak continues. After completing the procedure, the tongue depressor should be disposed properly in biohazard container. The swab should be placed immediately into the same VTM and shaft needs to be broken.^4,13^

## SPECIMEN PACKAGING

The sample should be safely packed in a triple-packing system which consists of three layers. For the first layer, the plastic pouch which the VTM came in, can be used. The labeled VTM should be placed inside the pouch and sealed with adhesive tape. If the cover is not available, a small-size zip-lock bag should be used. The packed VTM should be placed inside another zip-lock bag which acts as the second layer. Finally, the package should be inserted into a solid, unbreakable, leak-proof outer container which severs as the third layer. The VTM, zip-lock bag and the outer container should be labelled properly.^14^

## SPECIMEN TRANSPORTATION

The specimen should be transferred to the laboratory maintaining cold chain (2-4°C) throughout. The packed specimen should be placed inside the ice-box; two ice-pads should be placed on both sides of the container. The ice-box should be cleaned thoroughly outside with 1 % sodium hypochlorite and transferred to the laboratory as soon as possible with prior communication. If there is delay of more than 72 hours, sample should be stored at -70°C. Specimen data forms, letters, and other types of information that identify or describe the specimen for testing of SARS-COV-2 should be carried separately.^4,8,13^

## Conflict of Interest

**None.**
